# Calciprotein Particles and Serum Calcification Propensity: Hallmarks of Vascular Calcifications in Patients with Chronic Kidney Disease

**DOI:** 10.3390/jcm9051287

**Published:** 2020-04-29

**Authors:** Ciprian N. Silaghi, Tamás Ilyés, Adriana J. Van Ballegooijen, Alexandra M. Crăciun

**Affiliations:** 1 Department of Molecular Sciences, University of Medicine and Pharmacy “Iuliu Hațieganu”, 400012 Cluj-Napoca, Romania; tamasilyes94@gmail.com (T.I.); acraciun@umfcluj.ro (A.M.C.); 2 Department of Nephrology & Epidemiology and Biostatistics, Amsterdam UMC, location VUmc, 1117 HV Amsterdam, Netherlands; aj.vanballegooijen@vumc.nl

**Keywords:** calciprotein particles, calcification propensity, chronic kidney disease, vascular calcification

## Abstract

Cardiovascular complications are one of the leading causes of mortality worldwide and are strongly associated with atherosclerosis and vascular calcification (VC). Patients with chronic kidney disease (CKD) have a higher prevalence of VC as renal function declines, which will result in increased mortality. Serum calciprotein particles (CPPs) are colloidal nanoparticles that have a prominent role in the initiation and progression of VC. The T_50_ test is a novel test that measures the conversion of primary to secondary calciprotein particles indicating the tendency of serum to calcify. Therefore, we accomplished a comprehensive review as the first integrated approach to clarify fundamental aspects that influence serum CPP levels and T_50_, and to explore the effects of CPP and calcification propensity on various chronic disease outcomes. In addition, new topics were raised regarding possible clinical uses of T_50_ in the assessment of VC, particularly in patients with CKD, including possible opportunities in VC management. The relationships between serum calcification propensity and cardiovascular and all-cause mortality were also addressed. The review is the outcome of a comprehensive search on available literature and could open new directions to control VC.

## 1. Introduction

Serum calciprotein particles (CPPs) are colloidal nanoparticles comprising a combination of proteins (mainly fetuin-A, but also albumin and Gla-rich protein (GRP)) and calcium (Ca^2+^) containing compounds, primarily calcium phosphate [1,2,3]. They are first formed by the binding of Ca^2+^ precursors to the acidic residues of fetuin-A, a glycoprotein secreted by the liver [1,4]. These calcium–protein complexes, also known as calciprotein monomers, pass through further aggregation and maturation, resulting in primary calciprotein particles (CPP I) and later on, secondary calciprotein particles (CPP II) [5,6,7]. CPP I are small spherical colloidal nanoparticles that contain amorphous calcium phosphate, while CPP II contain crystalline calcium phosphate at their core, are larger than CPP I, and have a needle-shaped structure. This transition from CPP I to CPP II is called “ripening” and is hypothesized to be attributed to a reorganization of the colloidal nanoparticles into a more stable form [5]. The ripening process is influenced by a number of factors such as the concentration of fetuin-A, Ca^2+^, magnesium (Mg^2+^), phosphate (Pi), as well as the temperature and pH of the surrounding microenvironment [1,6,8].

The transition from CPP I to CPP II, which takes place naturally in serum, can also be induced in vitro, and the time needed for the transition to take place can be measured. Half of the time needed for the spontaneous transition from CPP I to CPP II, designated as T_50_, has been established as a strong predictor of the calcifying properties of serum [9]. A higher T_50_ is beneficial since serum with a higher T_50_ is less prone to calcify tissues compared to serum that has a lower T_50_.

Vascular calcification (VC) results in the thickening and increased rigidity of muscular arterial walls [10]. This is the consequence of two main types of calcification: intimal and medial calcification. Intimal calcification is associated with atherosclerosis, Ca^2+^ being deposited along with lipoproteins as well as phospholipids [11,12]. Medial calcification, which is more prevalent in chronic kidney disease (CKD), is the result of an osteogenic process similar to intramembranous ossification, which is independent of atherosclerosis and causes a decrease in compliance of the vessel wall [13,14,15]. Medial calcification occurs earlier in CKD patients compared to the general population [16].

With respect to CPPs in general and T_50_ in particular, there have been no reviews published until now that summarize findings related to both CPPs and T_50_. Therefore, the purpose of this review was to offer a synopsis of all studies published on CPPs and T_50_, respectively. We also aim to analyse and discuss their roles and clinical significance in patients prone to developing VC, as well as to establish possible new directions in the management of VC.

## 2. Methodology

### 2.1. Search Strategy

All databases that could be accessed through the PubMed search engine were selected for this review. Human, animal, and in vitro studies were all taken into account. Due to the specific nature of the selected domain and the fact that the majority of research papers were published relatively recently, the period of publication was not limited. A set of search terms was selected as follows: “Calciprotein particles”, “T_50_ AND calcification”, “Serum calcification propensity”. The search was performed in PubMed on the 4th of January 2020 for both search strings, yielding a total of 162 studies (78, 30, and 54 results, respectively). The results of the searches were organized into lists that were cross-checked between search terms, with duplicates being eliminated. After the initial screening of titles and abstracts, full-text articles were obtained for all eligible studies.

### 2.2. Selection, Screening, and Inclusion 

The authors jointly selected the inclusion and exclusion criteria. Only articles with abstracts were selected for screening, written in English including human, animal, and in vitro studies.

Studies that did not address CPPs and/or T_50_ in a medically relevant manner, such as physical or chemical characterization of CPPs, and studies that lacked a clear definition of methods and materials were not included. Reviews and case reports were excluded as well.

**Figure 1:**
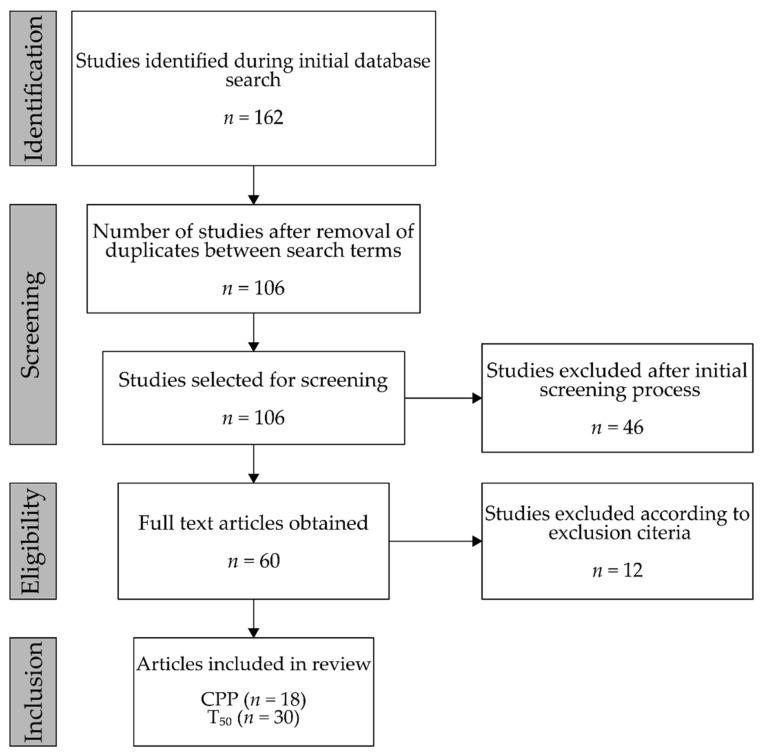
Flow diagram for the identification, selection, screening, and inclusion process. Abbreviations: CPP, calciprotein particles.

The identification, selection, screening, and inclusion process is summarized in Figure 1. After cross-checking and eliminating duplicates, the results of the search string “Serum calcification propensity” yielded three studies that were subsequently included in the same category as T_50_. In total, 18 studies were included for CPPs [3,17,18,19,20,21,22,23,24,25,26,27,28,29,30,31,32,33] and 30, including the aforementioned 3 studies, for T_50_ [34,35,36,37,38,39,40,41,42,43,44,45,46,47,48,49,50,51,52,53,54,55,56,57,58,59,60,61,62,63]. 

## 3. Molecular Background

### 3.1. Fetuin-A and Calciprotein Particles

**Figure 2:**
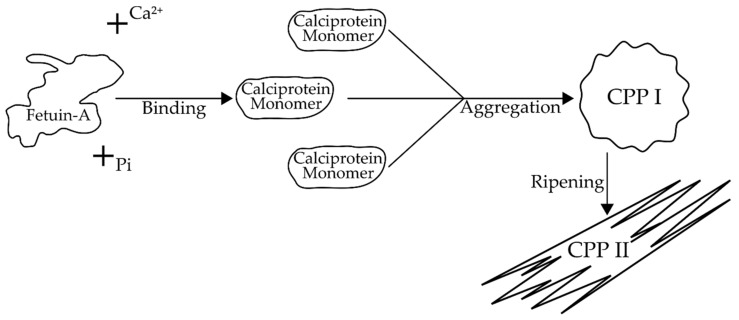
Fetuin-A transformation into CPP II. Abbreviations: Pi, phosphate; CPP I, primary calciprotein particle; CPP II, secondary calciprotein particle.

While CPPs contain a number of proteins that can bind Ca^2+^, e.g., Gla-rich protein (GRP) [3], as well as other serum proteins and lipoproteins such as albumin and apolipoprotein A1 [2], the main protein within the CPP structure is fetuin-A, also known as alpha-2-HS-glycoprotein. It is a 55–60 kDa glycoprotein, synthetized and secreted by the liver, which undergoes post-translational modifications, including phosphorylation [4,64]. While phosphorylation is crucial for its various interactions, e.g., with the insulin receptor, it is not required for mineral binding due to the number of acidic residues [1,4,65,66]. Each molecule of fetuin-A can bind up to 6 Ca^2+^ ions [67]. Calcium and Pi bound by fetuin-A form protein–mineral complexes called calciprotein monomers, the aggregation of which results in the formation of plasma-soluble amorphous colloidal particles, referred to as CPP I. The CPP I, which is spherical in nature and has a diameter of around 75 nm, circulates in plasma and eventually undergoes rearrangement into CPP II, which is more dense, with a larger diameter (120 nm), insoluble in serum, and has a needle-shaped crystalline structure [1]. This transition from the primary, more instable form, to the secondary, more stable form, is dubbed “ripening” [5]. The process is illustrated in Figure 2.

CPP I and CPP II are cleared by macrophages, especially Kupffer cells in the liver, thereby preventing tissular deposition of Ca^2+^ and Pi [68]. Studies have shown that CPP II induces vascular smooth muscle cell (VSMC) calcification in vitro, as well as the secretion of tumour necrosis factor α (TNF-α) in macrophages, while CPP I does not. CPP II was found to increase bone morphogenetic protein-2 as well as nuclear factor kappa-B expression in VSMCs. The calcification of VSMCs was also shown to be the result of the cellular uptake of CPP II, with CPP II being detected intracellularly in calcified VSMCs [29]. Both CPP I and CPP II were found to induce VSMC intimal hyperplasia, which was more pronounced in the case of CPP II [18]. Moreover, CPPs were found to induce secretion of interleukin 1β (IL-1β) in macrophages, however, to a lesser degree than hydroxyapatite crystals [31]. While both forms of CPP have pro-inflammatory effects, it is still less prominent than crystalline hydroxyapatite. The more pronounced pro-inflammatory effect of CPP II compared to that of CPP I might be attributed to its content of hydroxyapatite in crystalline form.

The CPPs are detected and quantified in serum indirectly, by assessing the fetuin-A levels via enzyme-linked immunosorbent assay (ELISA), before and after a high-speed centrifugation that precipitates all CPPs as CPP II. The difference between fetuin-A concentrations before and after centrifugation is interpreted as the amount of CPPs in the serum sample [33,69]. Because this method induces the ripening process before measuring CPP content, it only brings information regarding the total concentration of CPPs, without differentiating between CPP I and CPP II. To measure CPP I and CPP II concentrations independently, a flow-cytometry method can be used [70].

### 3.2. Calcifying Properties of Serum

A method for measuring the calcification inhibition capacity of serum was elaborated by Ismail et al. [71] based on electrochemical impedance. A prototype probe was successfully used to measure the impedance of a test solution consisting of bovine albumin, Ca^2+^, and Pi. Upon the addition of a calcification inhibitor, in that case fetuin-A, the electrical impedance of the solution would increase proportionately to the Ca^2+^ content, due to the inhibitor consuming Ca^2+^ ions by forming CPP I. Thus, the calcification inhibition capacity of the serum could be determined by measuring the variation of impedance of a solution containing Ca^2+^ and Pi in a known concentration, after the addition of serum.

**Figure 3:**
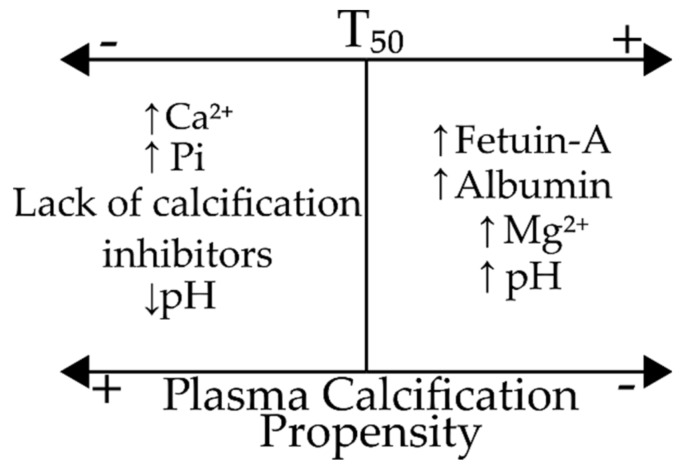
Effects of various factors upon T_50_ (half of the time needed for the spontaneous transition from CPP I to CPP II) and plasma calcification propensity. Abbreviations: Pi, phosphate; Ca^2+^, calcium; Mg^2+^, magnesium.

Pasch et al. [9] were the first to develop a plate-based nephelometric assay to measure the time needed for the transition from CPP I to CPP II in serum treated with Ca^2+^ and Pi solutions, and proposed the use of one half of the transition time to maximum turbidity, also known as T_50_, as a parameter to describe the calcifying properties of serum. The influence of factors such as pH and concentrations of various serum constituents upon T_50_ was also analysed, and is summarized in Figure 3.

## 4. Results

### 4.1. Calciprotein Particles

Human studies on serum CPP levels are summarized in Table 1, animal and in vitro studies on CPP are summarized in Table 2. The majority of studies used detection methods that did not differentiate between the two types of CPP. To avoid confusion, we used the term total CPP (tCPP) when referring to studies that did not specify the type of CPP analysed.

Dialysate from haemodialysis (HD) patients was found to contain CPP, and higher dialysate Ca^2+^ content was found to be associated with higher CPP concentration [24,30]. This suggests that CPP can be cleared from the plasma of patients with chronic kidney disease (CKD) through HD. In addition, CPP were found to induce VSMC calcification and intimal hyperplasia, with higher serum levels of CPP being associated with increased aortic stiffness [18,26,33]. CPP also induced the secretion of TNF-α and IL-1β in macrophages, with a more pronounced effect being attributed to CPP II. This pro-inflammatory response, however, was still inferior to that induced by pure hydroxyapatite crystals [29,31]. 

### 4.2. Calcification Propensity

Observational studies of T_50_ and outcomes are summarized in Table 3, and human intervention studies are summarized in Table 4. The majority of studies included in this section concern T_50_ in CKD and/or kidney transplant patients.

Oral Mg^2+^ supplementation, as well as increased Mg^2+^ concentration in dialysis solution was found to increase T_50_ in CKD patients [39,45,51]. The T_50_ was also found to be associated with serum Mg^2+^ levels in CKD patients, but not with eGFR [50]. Serum Mg^2+^ levels were directly associated with T_50_, which suggests that both oral Mg^2+^ supplementation, as well as increasing the Mg^2+^ content of dialysis solution could be a viable method to counterbalance VC to some extent in CKD patients. The use of citrate-buffered dialysis solution was found to significantly increase T_50_ as opposed to standard acetate-buffered dialysis solution in HD patients [34,46]. While platelet derived growth factor B hypomorphic animal brains showed signs of calcification, T_50_ did not differ compared to controls [61]. 

Lower T_50_ levels were also found to be associated with lower tissue oxygenation, as well as an increase in all-cause and cardiovascular mortality, especially in CKD and kidney transplant patients [36,42,48,49,52,56,57,60].

## 5. Discussion

This comprehensive review showed that multiple lines of evidence (cell, animal, and human) indicate that T_50_ is shorter in CKD and dialysis populations. A large amount of studies indicate that a lower T_50_ is related to VC, cardiovascular events, and mortality. These findings are robust across various populations and open up new directions to modify VC especially in patients with CKD. One of these factors that can influence the tendency to calcify is Mg^2+^. Oral Mg^2+^ supplementation as well as increased dialysis solution Mg^2+^ concentration had beneficial effects on T_50_ [39,45,52], and a lower T_50_ was associated with cardiovascular and all-cause mortality in various populations [36,42,49,52,56,57,60]. It is worth noting the correlation between higher serum CPP content, especially CPP II, and VSMC inflammation as well as calcification [18,26,29,31,33]. Taking the included studies into consideration, we address two topics for further research in this relatively recent domain.

### 5.1. The Effect of Dialysis Solution Composition upon Serum Calcification Propensity in CKD Patients

The transition from CPP I to CPP II is delayed by the presence of Mg^2+^, this effect being dependent upon the concentration of Mg^2+^. The presence of Mg^2+^, however, does not inhibit VSMC calcification in the presence of CPP II, suggesting that the anti-calcific effects of Mg^2+^ are more related to preventing the transition from CPP I to CPP II [19]. This would also explain the effect of Mg^2+^ upon increasing T_50_. However, the exact mechanism by which Mg^2+^ inhibits the maturation of CPP I is not completely understood. One possible mechanism might lie in the ability of Mg^2+^ to inhibit Ca^2+^ and Pi crystallization [72], which is a necessary step in CPP maturation.

Studies suggest that there is a significant amount of CPPs in the dialysate of CKD patients on peritoneal dialysis. That CPP content was also directly proportional to the dialysate’s Ca^2+^ content [24]. While HD was found to increase T_50_, thus reducing the calcification propensity of the patient’s plasma [37,54], serum CPP I and CPP II levels seem to be unaffected by standard HD [17]. 

First of all, this would suggest that the increase in T_50_ after initiation of HD is not attributed to the clearance of CPPs per se, but to the reduction of factors that precipitate the ripening process, most probably the reduction of Ca^2+^ and Pi. Second of all, CPPs, while not being cleared from the serum under standard HD conditions, are cleared by peritoneal dialysis to some degree. However, if the Mg^2+^ concentration of HD dialysis solution is increased, CPPs appear to pass the dialysis membrane and are cleared from the patient’s serum [17]. This would, in part, explain the significant increase of T_50_ in patients treated with a dialysis solution containing a larger Mg^2+^ concentration compared to standard solution [45].

In addition to the beneficial effect of increased Mg^2+^ content in dialysis solution upon the serum calcification propensity in CKD patients, the use of an acetate-free, citrate-acidified dialysis solution was also found to increase T_50_ thus reducing the calcification propensity [34,46].

**Figure 4:**
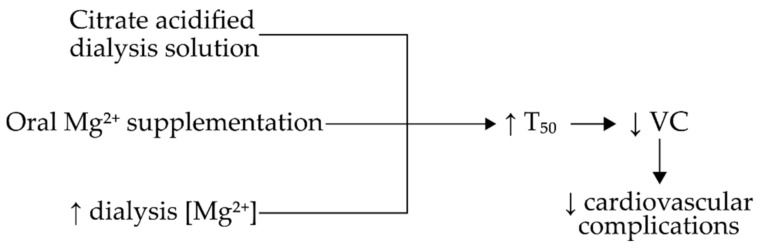
Summary of factors that increase T_50_ in CKD patients. Abbreviations: Mg, magnesium; VC, vascular calcification.

Patients with CKD who received oral Mg^2+^ supplementation showed a significant increase in T_50_ [39,51]. In post-menopausal women, the introduction of oral Ca^2+^ supplementation showed a decrease in T_50_, however, this decrease did not differ significantly from the control group [55]. These observations correspond with the findings of Pasch et al. [9], who determined that higher serum Mg^2+^ levels will increase T_50_. A summary of the aforementioned factors upon T_50_ is presented in Figure 4.

Furthermore, it is well known that patients with CKD have a significantly higher risk for VC and associated cardiovascular mortality [73]. Developing a standardized treatment plan for end-stage CKD patients on HD or peritoneal dialysis that would take into account the above outlined criteria, namely the increased Mg^2+^ content of dialysis solution and the use of citrate instead of acetate, should be validated and subsequently introduced into a therapeutic protocol. Patients with HD, as well as those with CKD who do not require HD, could also benefit from a reduction in oral Ca^2+^ and an increase in oral Mg^2+^ supplementation, respectively. Such an approach to the management of VC and the possible ensuing reduction of cardiovascular mortality rates in CKD patients could lead to an increased quality of life, especially for patients undergoing HD or peritoneal dialysis, delaying the onset or decreasing the severity of cardiovascular complications associated with CKD.

### 5.2. The T_50_ Test Could Be Used as a Factor in the Staging and/or Prognosis of CKD

There are plentiful studies, conducted on large sample sizes, that came to the conclusion that lower T_50_ corresponding to higher calcification propensity is strongly associated with increased cardiovascular and all-cause mortality rates [36,42,49,52,56,57,60]. Lower T_50_ was also associated with coronary artery calcification progression as well as greater risk for cardiovascular disease outcomes, such as myocardial infarction and peripheral vascular events [38,42,49].

The investigation of a possible association between T_50_ and eGFR could lead to the development of a reference interval for T_50_ in CKD patients, which is dependent on CKD stage. Such a reference interval, which has not yet been established, could be used as an additional prognostic parameter for CKD patients, especially those undergoing HD or peritoneal dialysis treatment. There was conflicting evidence that links serum CPP levels and T_50_ to eGFR. Yamada et al. [28] found that CPP levels were inversely associated with eGFR in diabetic patients. However, that study was conducted on diabetic patients, not CKD patients, and the patient group was relatively small as well. On the other hand, Bielesz et al. [50], found that T_50_ was not associated with eGFR in CKD stage I–V patients, instead being associated with numerous parameters, including Pi and Mg^2+^ levels. A similar result was obtained by de Seigneux et al. [59], who discovered that T_50_ was independent of eGFR in kidney transplant donors, which could be attributed to the compensation effect of an otherwise healthy remaining kidney. Those studies clearly pointed that while serum CPP levels are correlated with eGFR, T_50_ was not.

The CPP levels and T_50_ do not seem to be directly correlated with one another, although T_50_ is greatly influenced by serum Ca^2+^ levels and, in addition, CPP levels are directly proportional to circulating Ca^2+^ levels. Considering the previously discussed ideas, it could be hypothesized that CPP levels are correlated with T_50_, justifying further studies in larger populations to investigate the association between T_50_ and eGFR. However, until the completion of this review, no studies have identified this relationship.

Even in the absence of a link between T_50_ and eGFR, but in the context of association between higher serum calcification propensity and increased cardiovascular and all-cause mortality rates especially in CKD patients, the use of T_50_ as risk factor that can be monitored should be considered. The ensuing introduction of measures to decrease calcification propensity could significantly reduce VC and related mortality in CKD patients.

An interesting opportunity would be to expand the area of research towards the involvement of CPPs in the calcification paradox, in which the presence of vascular calcification overlaps at the same time with bone demineralization assessed by a decrease in bone mineral density (BMD) [74]. It is difficult to decode how CPP and the interplay between vasculature–bone–kidney underlie the deleterious effect of calcification. On one hand, fetuin-A accumulates in calcified atherosclerotic plaques [75], but also in bone where it inhibits mineralization and halts bone matrix protein expression [76]. On the other hand, serum levels of fetuin-A were found to be decreased in patients with end-stage renal disease [77]. Contrariwise, serum CPP increases in patients with CKD III–IV, with it being the highest in HD patients [32] but with less fetuin-A content as CKD stage worsens [3]. Probably the turn-over of CPP is accelerated in CKD patients, but fetuin-A is consumed exerting its systemic anti-calcification effect necessary to counteract VC as CKD stage aggravates.

In addition to the well-known presence of VC in patients with CKD, an important decrease of BMD was also reported [78]. In maintenance HD patients, serum fetuin-A was inversely associated with coronary artery calcification and positively with BMD [79]. In respect to VC, serum CPP appears to behave divergently regarding fetuin-A dynamics in CKD patients: higher CPP levels are associated with increased aortic stiffness [33] and larger CPP II diameters were found in patients with VC [23]. As might be expected, T_50_ was inversely associated with coronary artery calcification (CAC) severity in CKD patients [38], thereby, the T_50_ test seems to mimic serum fetuin-A variations in respect to VC, as they were found to be associated [50]. Regarding the loss of skeletal mineral, T_50_ was not associated with BMD [38] and in the case of CPPs we did not find conclusive studies. To make the puzzle even more complicated, we could introduce the relationship between CPPs or T_50_ and eGFR, as discussed above. Thereby, CPPs were found to be inversely correlated with eGFR [28], instead of T_50_, which was independent of eGFR [50,59].

However, an attempt to explain the paradox of calcification on the vasculature–bone–kidney axis only in terms of fetuin-A content of CPPs is an exercise of simplification. Given this standpoint, more targeted studies are needed to demonstrate that CPPs are more likely to hold the key on how physiological ossification has correspondence with pathological calcification.

Nevertheless, we need to take into account the current limitations of the T_50_ test. Several weaknesses were identified by Pasch et al. [9]: the test overrides the contribution of VSMCs and calcifying myeloid cells in promoting VC in vivo, and the serum pH had no influence on the test due to the presence of a strong buffer. Another issue is attaining standardized conditions to perform the test. Consequently, even if a reference interval would be preferable to be established, each laboratory is likely to set up its own different reference interval, hence it is hard to envisage an accepted consensus interval. The test is robust but needs further development in terms of time per test which is too long to be clinically implemented for the moment: to perform a 96-well format takes 10 h [9].

In addition, simply minimizing the T_50_ as a marker only for VC may be incomplete. The T_50_ could be considered as a momentary status of the sum of pro- and anti-calcification factors in the serum of a patient, but this may have implications on other pathophysiological processes, thus opening a wide field of research. Accordingly, the term mineral stress has been coined by Pasch et al. [80] and refers to the interaction between inflammation, oxidative stress, and calcification promoted by CPP II.

## 6. Conclusions

The relatively recent discovery of CPPs opens up new possibilities for the prevention of VC and the attempt to quantify the serum calcification propensity via T_50_. Even though the factors that influence serum CPP levels, including their ripening process, as well the effect of various factors upon T_50_ and its variation in different diseases is incompletely understood, there is mounting evidence suggesting that T_50_ could be a viable marker in the assessment of VC. Moreover, T_50_ could be valuable in managing VC in CKD patients, especially those undergoing HD, who have a significantly increased risk for developing cardiovascular complications. In these situations, the early introduction of a treatment strategy that increases T_50_ could mitigate the obvious complications related to VC. Such an approach is still at an early phase, warranting future studies on the use of T_50_ as a standard tool in the assessment of VC, thus allowing early measures to prevent cardiovascular complications in patients at risk.

## Abbreviations:

BMDBone Mineral DensityCa^2+^CalciumCACCoronary Artery CalcificationCKDChronic Kidney DiseaseCPP IPrimary Calciprotein ParticlesCPP IISecondary Calciprotein ParticlesCPPCalciprotein ParticlesCVDCardiovascular DiseaseCVECardiovascular EventeGFREstimated Glomerular Filtration RateELISAEnzyme-Linked Immunosorbent Assay GRPGla-Rich ProteinHDHaemodialysisH_2_SHydrogen SulphideIL-1βInterleukin 1βMg^2+^MagnesiumN/ANot ApplicablePiPhosphateSLESystemic Lupus ErythematosustCPPTotal Calciprotein ParticlesTNF-αTumour Necrosis Factor αTODTime of DeathVCVascular CalcificationVSMCVascular Smooth Muscle Cell

## Figures and Tables

**Table 1:** Summary of 11 human studies on calciprotein particle (CPP).

Author, Year	Study Design, Duration	Number of Subjects, Disease	CPP Type Studied	Findings
Nakazato et al. 2019 [20]	cross-sectional, N/A	71 ACS	tCPP	High CPP levels associated with atherosclerosis.
Chen et al. 2019 [23]	cross-sectional, N/A	45 CKD stage IV–V	CPP II	Larger CPP II diameter in patients with VC.
Viegas et al. 2018 [3]	cross-sectional, N/A	16 CKD stage II-IV, 20 CKD stage V	tCPP	CPP from CKD stage V patients contained less fetuin-A and GRP and had CPP II like characteristics.
Yamada et al. 2018 [28]	cross-sectional, N/A	10 diabetes mellitus type 2	tCPP	CPP elevated 2 h post-meal, CPP inversely correlated with eGFR.
Cai et al. 2015 [30]	cross-sectional, N/A	20 peritoneal dialysis	tCPP	CPP present, fetuin-A abundant in peritoneal dialysis effluent.
Smith et al. 2013 [32]	cross-sectional, N/A	11 CKD stage III–IV, 42 HD, 18 peritoneal dialysis, 13 chronic inflammatory disease	tCPP	CPP increased in CKD III-IV, HD, peritoneal dialysis and chronic inflammatory disease patients; CPP was highest in HD patients with calcific uremic arteriolopathy.
Smith et al. 2012 [33]	cross-sectional, N/A	200 CKD stage III–IV	tCPP	Higher CPP levels associated with increased aortic stiffness.
Cai et al. 2018 [24]	prospective cohort, 7 weeks	12 peritoneal dialysis	tCPP	Dialysate with higher Ca^2+^ concentration had higher CPP content.
Ruderman et al. 2018 [25]	prospective cohort, 12 months	62 HD	CPP I	Increase of serum CPP I after cessation of cinacalcet treatment.
Bressendorff et al. 2019 [17]	Interventional, 28 days	57 HD	CPP I, CPP II	Higher Mg^2+^ concentration dialysis solution reduced both CPP I and CPP II levels, compared to standard dialysis solution.
Nakamura et al. 2019 [21]	Interventional, 16 weeks	24 HD	tCPP	Lower CPP in lanthanum carbonate treated patients vs. calcium carbonate.

Abbreviations: HD, haemodialysis; ACS, acute coronary syndrome; CPP, calciprotein particle; CPP I, primary calciprotein particle; CPP II, secondary calciprotein particle; tCPP, total calciprotein particles; CKD, chronic kidney disease; VC, vascular calcification; GRP, Gla-rich protein; eGFR, estimated glomerular filtration rate; N/A, not applicable.

**Table 2:** Summary of 1 animal and 6 in vitro studies on CPP.

Author, Year	Study Design	Animals/Cells	CPP Type Studied	Findings
Nemoto et al. 2019 [22]	animal	rats with 5/6 nephrectomy	tCPP	Lower CPP in rats treated with sucroferric oxyhydroxide.
Shishkova et al. 2019 [18]	in vitro	VSMCs	CPP I, CPP II	Both CPP I and CPP II induced VSMC intimal hyperplasia, more pronounced in case of CPP II.
Ter Braake et al. 2019 [19]	in vitro	VSMCs	CPP II	CPP II induced VSMC calcification.
Aghagolzadeh et al. 2017 [26]	in vitro	VSMCs	tCPP	H_2_S inhibits CPP induced VSMC calcification.
Cai et al. 2017 [27]	in vitro	VSMCs	CPP II	Pi or CPP II alone did not initiate VSMC mineralization, but CPP II with Pi did.
Aghagolzadeh et al. 2016 [29]	in vitro	VSMCs	CPP I, CPP II	CPP II induced calcification in VSMCs, CPP I did not.
Smith et al. 2013 [31]	in vitro	VSMCs	tCPP	CPP induce secretion of TNF-α and IL-1β in macrophages, but less significantly than that induced by hydroxyapatite crystals.

Abbreviations: VSMCs, vascular smooth muscle cells; CPP, calciprotein particle; CPP I, primary calciprotein particle; CPP II, secondary calciprotein particle; tCPP, total calciprotein particles; H_2_S, hydrogen sulphide; Pi, phosphate; TNF-α, tumour necrosis factor α; IL-1β, interleukin 1β.

**Table 3:** Summary of 18 observational studies on T_50_ and health outcomes.

Author, Year	Study Design	Follow-Up Time	Number of Subjects, Disease	Findings
Bullen et al. 2019 [41]	cross-sectional	N/A	149 men with osteoporosis	T_50_ was not associated with bone mineral density.
Dahdal et al. 2018 [47]	cross-sectional	N/A	168, SLE	T_50_ was negatively associated with disease activity.
Pruijm et al. 2017 [48]	cross-sectional	N/A	58, CKD; 48, hypertension	Lower T_50_ was associated with reduced tissue oxygenation and perfusion.
Bielesz et al. 2017 [50]	cross-sectional	N/A	118, CKD stage I–V	T_50_ associated with Pi, Mg^2+^ and fetuin-A but not with eGFR.
Dekker et al. 2016 [54]	cross-sectional	N/A	64, HD	T_50_ increased post-haemodialysis and post-haemodiafiltration.
Voelkl et al. 2018 [63]	cross-sectional	N/A	16, CKD; 20, HD	T_50_ was lower in CKD patients compared to controls.
van Dijk et al. 2019 [35]	prospective cohort	15 years	216, type 1 diabetes	T_50_ not associated with mortality.
Bundy et al. 2019 [36]	prospective cohort	At TOD or 11.2 years	3404, CKD stage II–IV	Lower T_50_ associated with cardiovascular events and all-cause mortality.
Ponte et al. 2019 [37]	prospective cohort	3 months	46, HD; 12, peritoneal dialysis	Higher T _50_ after dialysis initiation.
Bundy et al. 2019 [38]	prospective cohort	3.2 ± 0.6 years	780, CKD stage II–IV	Lower T_50_ was associated with greater CAC severity and progression, however, T_50_ was not associated with CAC incidence.
Bostom et al. 2018 [42]	prospective cohort	median of 2.18 years	685, CVD	Lower T_50_ and fetuin-A levels were associated with greater risk for CVD outcomes.
Pasch et al. 2017 [49]	prospective cohort	At TOD or first non-fatal CVE	2785, HD	Lower T_50_ associated with all-cause mortality, myocardial infarction, and peripheral vascular events.
Lorenz et al. 2017 [52]	prospective cohort	24 months	188, HD	T_50_ rate of decline significantly predicted all-cause and cardiovascular mortality.
Dahle et al. 2016 [56]	prospective cohort	median of 5.1 years	1435, kidney transplant	Lower T_50_ associated with all-cause and cardiac mortality.
Keyzer et al. 2016 [57]	prospective cohort	median of 3.1 years	699, kidney transplant	Lower T_50_ associated with increased graft failure, all-cause, and cardiac mortality.
de Seigneux et al. 2015 [59]	prospective cohort	1 year	21, kidney donors	T_50_ was independent of eGFR.
Smith et al. 2014 [60]	prospective cohort	median of 5.3 years	184, CKD stage III–IV	Lower T_50_ associated with higher all-cause mortality.
Berchtold et al. 2016 [58]	retrospective cohort	between 2 and 43 years	129, kidney transplant	T_50_ associated with interstitial fibrosis and vascular lesions.

Abbreviations: SLE, systemic lupus erythematosus; HD, haemodialysis; CKD, chronic kidney disease; CAC, coronary artery calcification; CVD, cardiovascular disease; Mg^2+^, magnesium; TOD, time of death; CVE, cardiovascular event; Pi, phosphate; eGFR, estimated glomerular filtration rate; N/A, not applicable.

**Table 4:** Summary of 11 human interventional studies on T_50_ with health outcomes.

Author, Year	Study Duration	Number of Subjects, Disease	Findings
Smerud et al. 2017 [53]	1 year	123, kidney transplant	T_50_ increased with no further change after 1 year, ibandronate had no effect on T_50_.
Andrews et al. 2018 [43]	12 weeks	80, CKD with hyperuricemia	Allopurinol lowered uric acid levels but had no effect on T_50_.
Lorenz et al. 2018 [46]	3 months	78, HD	Acetate-free, citrate-acidified, standard bicarbonate dialysis solution increased T_50_ compared to acetate dialysis solution.
Ussif et al. 2018 [44]	1 year	76, kidney transplant	Paricalcitol supplementation had no effect on T_50_.
Bressendorff et al. 2018 [45]	28 days	57, HD	Higher dialysis solution Mg^2+^ concentration increased T_50_.
Bristow et al. 2016 [55]	3 months	41, post-menopausal women	Insignificant decrease of T_50_ in the group treated with oral calcium carbonate supplement.
Bressendorff et al. 2017 [51]	8 weeks	36, CKD III–IV	Oral Mg^2+^ supplementation increased T_50_.
Aigner et al. 2019 [40]	4 weeks	35, CKD	Oral bicarbonate supplementation showed no effect on T_50_ in acidotic CKD patients.
Kendrick et al. 2018 [62]	14 weeks	18, CKD	Oral sodium bicarbonate supplementation showed no effect on T_50_ in CKD patients with low serum bicarbonate levels.
Ter Meulen et al. 2019 [34]	2 weeks	18, HD	Citric acid-buffered dialysis solution increased T_50_ compared to acetate-buffered solution.
Quiñones et al. 2019 [39]	2 weeks	9, CKD stage III, 9, CKD stage V	Effervescent, oral, calcium-magnesium citrate increased T_50_.

Abbreviations: HD, haemodialysis; CKD, chronic kidney disease; Mg^2+^, magnesium.

## References

[1] Heiss A., Duchesne A., Denecke B., Grötzinger J., Yamamoto K., Renné T., Jahnen-Dechent W. (2003). Structural Basis of Calcification Inhibition by α2-HS Glycoprotein/Fetuin-A. J. Boil. Chem..

[2] Köppert S., Büscher A., Babler A., Ghallab A., Buhl E.M., Latz E., Hengstler J.G., Smith E.R., Jahnen-Dechent W. (2018). Cellular Clearance and Biological Activity of Calciprotein Particles Depend on Their Maturation State and Crystallinity. Front. Immunol..

[3] Viegas C.S., Santos L., Macedo A., Matos A.P., Silva A.P., Neves P.L., Staes A., Gevaert K., Morais R., Vermeer C. (2018). Chronic Kidney Disease Circulating Calciprotein Particles and Extracellular Vesicles Promote Vascular Calcification. Arter. Thromb. Vasc. Boil..

[4] Schinke T., Amendt C., Trindl A., Pöschke O., Müller-Esterl W., Jahnen-Dechent W. (1996). The Serum Protein α2-HS Glycoprotein/Fetuin Inhibits Apatite Formationin Vitroand in Mineralizing Calvaria Cells. J. Boil. Chem..

[5] Holt S.G., Smith E.R. (2016). Fetuin-A-containing calciprotein particles in mineral trafficking and vascular disease. Nephrol. Dial. Transplant..

[6] Heiss A., Eckert T., Aretz A., Richtering W., Van Dorp W., Schäfer C., Jahnen-Dechent W. (2008). Hierarchical Role of Fetuin-A and Acidic Serum Proteins in the Formation and Stabilization of Calcium Phosphate Particles. J. Boil. Chem..

[7] Cai M.M.X., Smith E.R., Holt S.G. (2015). The role of fetuin-A in mineral trafficking and deposition. BoneKey Rep..

[8] Rochette C.N., Rosenfeldt S., Heiss A., Narayanan T., Ballauff M., Jahnen-Dechent W. (2009). A Shielding Topology Stabilizes the Early Stage Protein-Mineral Complexes of Fetuin-A and Calcium Phosphate: A Time-Resolved Small-Angle X-ray Study. ChemBioChem.

[9] Pasch A., Farese S., Gräber S., Wald J., Richtering W., Floege J., Jahnen-Dechent W. (2012). Nanoparticle-Based Test Measures Overall Propensity for Calcification in Serum. J. Am. Soc. Nephrol..

[10] Hunt J.L., Fairman R., Mitchell M.E., Carpenter J.P., Golden M., Khalapyan T., Wolfe M., Neschis D., Milner R., Scoll B. (2002). Bone Formation in Carotid Plaques. Stroke.

[11] Van Oostrom O., Fledderus J.O., De Kleijn D., Pasterkamp G., Verhaar M. (2009). Smooth Muscle Progenitor Cells: Friend or Foe in Vascular Disease?. Curr. Stem Cell Res. Ther..

[12] Persy V., D’Haese P. (2009). Vascular calcification and bone disease: The calcification paradox. Trends Mol. Med..

[13] Ho C.Y., Shanahan C.M. (2016). Medial Arterial Calcification. Arter. Thromb. Vasc. Boil..

[14] Lanzer P., Boehm M., Sorribas V., Thiriet M., Janzen J., Zeller T., Hilaire C.S., Shanahan C.M. (2014). Medial vascular calcification revisited: Review and perspectives. Eur. Hear. J..

[15] Magne D., Julien M., Vinatier C., Weiss P., Guicheux J., Merhi-Soussi F. (2005). Cartilage formation in growth plate and arteries: From physiology to pathology. BioEssays.

[16] Goodman W.G., Goldin J., Kuizon B.D., Yoon C., Gales B., Sider D., Wang Y., Chung J., Emerick A., Greaser L. (2000). Coronary-Artery Calcification in Young Adults with End-Stage Renal Disease Who Are Undergoing Dialysis. New Engl. J. Med..

[17] Bressendorff I., Hansen D., Pasch A., Holt S.G., Schou M., Brandi L., Smith E.R. (2019). The effect of increasing dialysate magnesium on calciprotein particles, inflammation and bone markers: Post hoc analysis from a randomized controlled clinical trial. Nephrol. Dial. Transplant..

[18] Shishkova D., Velikanova E., Sinitsky M., Tsepokina A., Gruzdeva O.V., Bogdanov L., Kutikhin A. (2019). Calcium Phosphate Bions Cause Intimal Hyperplasia in Intact Aortas of Normolipidemic Rats through Endothelial Injury. Int. J. Mol. Sci..

[19] Ter Braake A.D., Eelderink C., Zeper L.W., Pasch A., Bakker S.J.L., De Borst M.H., Hoenderop J.G.J., De Baaij J.H. (2019). Calciprotein particle inhibition explains magnesium-mediated protection against vascular calcification. Nephrol. Dial. Transplant..

[20] Nakazato J., Hoshide S., Wake M., Miura Y., Kuro-O M., Kario K. (2019). Association of calciprotein particles measured by a new method with coronary artery plaque in patients with coronary artery disease: A cross-sectional study. J. Cardiol..

[21] Nakamura K., Nagata Y., Hiroyoshi T., Isoyama N., Fujikawa K., Miura Y., Matsuyama H., Kuro-O M. (2019). The effect of lanthanum carbonate on calciprotein particles in hemodialysis patients. Clin. Exp. Nephrol..

[22] Nemoto Y., Kumagai T., Ishizawa K., Miura Y., Shiraishi T., Morimoto C., Sakai K., Omizo H., Yamazaki O., Tamura Y. (2019). Phosphate binding by sucroferric oxyhydroxide ameliorates renal injury in the remnant kidney model. Sci. Rep..

[23] Chen W., Anokhina V., Dieudonne G., Abramowitz M.K., Kashyap R., Yan C., Wu T.T., Bentley K.L.D.M., Miller B.L., Bushinsky D. (2019). Patients with advanced chronic kidney disease and vascular calcification have a large hydrodynamic radius of secondary calciprotein particles. Nephrol. Dial. Transplant..

[24] Cai M.M.X., Smith E.R., Kent A., Huang L., Hewitson T., McMahon L.P., Holt S.G. (2018). Calciprotein Particle Formation in Peritoneal Dialysis Effluent is Dependent on Dialysate Calcium Concentration. Perit. Dial. Int..

[25] Ruderman I., Smith E.R., Toussaint N.D., Hewitson T., Holt S.G. (2018). Longitudinal changes in bone and mineral metabolism after cessation of cinacalcet in dialysis patients with secondary hyperparathyroidism. BMC Nephrol..

[26] Aghagolzadeh P., Radpour R., Bachtler M., Van Goor H., Smith E.R., Lister A., Odermatt A., Feelisch M., Pasch A. (2017). Hydrogen sulfide attenuates calcification of vascular smooth muscle cells via KEAP1/NRF2/NQO1 activation. Atherosclerosis.

[27] Cai M.M.X., Smith E.R., Tan S.-J., Hewitson T., Holt S.G. (2017). The Role of Secondary Calciprotein Particles in the Mineralisation Paradox of Chronic Kidney Disease. Calcif. Tissue Int..

[28] Yamada H., Kuro-O M., Ishikawa S.-E., Funazaki S., Kusaka I., Kakei M., Hara K. (2018). Daily variability in serum levels of calciprotein particles and their association with mineral metabolism parameters: A cross-sectional pilot study. Nephrology.

[29] Aghagolzadeh P., Bachtler M., Bijarnia R., Jackson C.B., Smith E.R., Odermatt A., Radpour R., Pasch A. (2016). Calcification of vascular smooth muscle cells is induced by secondary calciprotein particles and enhanced by tumor necrosis factor-α. Atherosclerosis.

[30] Cai M.M.X., Wigg B., Smith E.R., Hewitson T., McMahon L.P., Holt S.G. (2014). Relative abundance of fetuin- A in peritoneal dialysis effluent and its association with in situ formation of calciprotein particles: An observational pilot study. Nephrology.

[31] Smith E.R., Hanssen E., McMahon L.P., Holt S.G. (2013). Fetuin-A-Containing Calciprotein Particles Reduce Mineral Stress in the Macrophage. PLoS ONE.

[32] Smith E.R., Cai M.M., McMahon L.P., Pedagogos E., Toussaint N.D., Brumby C., Holt S.G. (2013). Serum fetuin-A concentration and fetuin-A-containing calciprotein particles in patients with chronic inflammatory disease and renal failure. Nephrology.

[33] Smith E.R., Ford M.L., Tomlinson L., Rajkumar C., McMahon L.P., Holt S.G. (2012). Phosphorylated fetuin-A-containing calciprotein particles are associated with aortic stiffness and a procalcific milieu in patients with pre-dialysis CKD. Nephrol. Dial. Transplant..

[34] Ter Meulen K.J., Dekker M.J.E., Pasch A., Broers N.J.H., Van Der Sande F.M., Kooman J.P., Konings C.J.A.M., Gsponer I.M., Bachtler M.D.N., Gauly A. (2019). Citric-acid dialysate improves the calcification propensity of hemodialysis patients: A multicenter prospective randomized cross-over trial. PLoS ONE.

[35] Van Dijk P.R., Hop H., Waanders F., Mulder U.J., Pasch A., Hillebrands J.-L., Van Goor H., Bilo H.J. (2019). Serum calcification propensity in type 1 diabetes associates with mineral stress. Diabetes Res. Clin. Pract..

[36] Bundy J.D., Cai X., Mehta R.C., Scialla J.J., De Boer I.H., Hsu C.-Y., Go A.S., Dobre M., Chen J., Rao P.S. (2019). Serum Calcification Propensity and Clinical Events in CKD. Clin. J. Am. Soc. Nephrol..

[37] Ponte B., Pruijm M., Pasch A., Dufey-Teso A., Martin P.-Y., De Seigneux S. (2019). Dialysis initiation improves calcification propensity. Nephrol. Dial. Transplant..

[38] Bundy J.D., Cai X., Scialla J.J., Dobre M.A., Chen J., Hsu C.-Y., Leonard M.B., Go A.S., Rao P.S., Lash J.P. (2019). Serum Calcification Propensity and Coronary Artery Calcification Among Patients With CKD: The CRIC (Chronic Renal Insufficiency Cohort) Study. Am. J. Kidney Dis..

[39] Quiñones H., Hamdi T., Sakhaee K., Pasch A., Moe O.W., Pak C.Y.C. (2018). Control of metabolic predisposition to cardiovascular complications of chronic kidney disease by effervescent calcium magnesium citrate: A feasibility study. J. Nephrol..

[40] Aigner C., Cejka D., Sliber C., Fraunschiel M., Sunder-Plassmann G., Gaggl M. (2019). Oral Sodium Bicarbonate Supplementation Does Not Affect Serum Calcification Propensity in Patients with Chronic Kidney Disease and Chronic Metabolic Acidosis. Kidney Blood Press. Res..

[41] Bullen A.L., Anderson C.A.M., Hooker E.R., Kado D.M., Orwoll E., Pasch A., Ix J.H. (2019). Correlates of T50 and relationships with bone mineral density in community-living older men: The osteoporotic fractures in men (MrOS) study. Osteoporos. Int..

[42] Bostom A., Pasch A., Madsen T., Roberts M.B., Franceschini N., Steubl D., Garimella P.S., Ix J.H., Tuttle K.R., Ivanova A. (2018). Serum Calcification Propensity and Fetuin-A: Biomarkers of Cardiovascular Disease in Kidney Transplant Recipients. Am. J. Nephrol..

[43] Andrews E.S., Perrenoud L., Nowak K.L., You Z., Pasch A., Chonchol M., Kendrick J., Jalal D. (2018). Examining the effects of uric acid-lowering on markers vascular of calcification and CKD-MBD. A post-hoc analysis of a randomized clinical trial. PLoS ONE.

[44] Ussif A.M., Pihlstrøm H., Pasch A., Holdaas H., Hartmann A., Smerud K., Åsberg A. (2018). Paricalcitol supplementation during the first year after kidney transplantation does not affect calcification propensity score. BMC Nephrol..

[45] Bressendorff I., Hansen D., Schou M., Pasch A., Brandi L. (2018). The Effect of Increasing Dialysate Magnesium on Serum Calcification Propensity in Subjects with End Stage Kidney Disease. Clin. J. Am. Soc. Nephrol..

[46] Lorenz G., Mayer C.C., Bachmann Q., Stryeck S., Braunisch M.C., Haller B., Carbajo-Lozoya J., Schmidt A., Witthauer S., Abuzahu J. (2018). Acetate-free, citrate-acidified bicarbonate dialysis improves serum calcification propensity—a preliminary study. Nephrol. Dial. Transplant..

[47] Dahdal S., Devetzis V., Chalikias G., Tziakas D., Chizzolini C., Ribi C., Trendelenburg M., Eisenberger U., Hauser T., Pasch A. (2018). Serum calcification propensity is independently associated with disease activity in systemic lupus erythematosus. PLoS ONE.

[48] Pruijm M., Lu Y., Megdiche F., Piskunowicz M., Milani B., Stuber M., Bachtler M., Vogt B., Burnier M., Pasch A. (2017). Serum calcification propensity is associated with renal tissue oxygenation and resistive index in patients with arterial hypertension or chronic kidney disease. J. Hypertens..

[49] Pasch A., Block G.A., Bachtler M., Smith E.R., Jahnen-Dechent W., Arampatzis S., Chertow G.M., Parfrey P., Ma X., Floege J. (2016). Blood Calcification Propensity, Cardiovascular Events, and Survival in Patients Receiving Hemodialysis in the EVOLVE Trial. Clin. J. Am. Soc. Nephrol..

[50] Bielesz B., Reiter T., Marculescu R., Gleiss A., Bojic M., Kieweg H., Cejka D. (2017). Calcification Propensity of Serum is Independent of Excretory Renal Function. Sci. Rep..

[51] Bressendorff I., Hansen D., Schou M., Silver B., Pasch A., Bouchelouche P., Pedersen L., Rasmussen L.M., Brandi L. (2016). Oral Magnesium Supplementation in Chronic Kidney Disease Stages 3 and 4: Efficacy, Safety, and Effect on Serum Calcification Propensity-A Prospective Randomized Double-Blinded Placebo-Controlled Clinical Trial. Kidney Int. Rep..

[52] Lorenz G., Steubl D., Kemmner S., Pasch A., Koch-Sembdner W., Pham D., Haller B., Bachmann Q., Mayer C.C., Wassertheurer S. (2017). Worsening calcification propensity precedes all-cause and cardiovascular mortality in haemodialyzed patients. Sci. Rep..

[53] Smerud K., Åsberg A., Kile H., Pasch A., Dahle D.O., Bollerslev J., Godang K., Hartmann A. (2017). A rapid and sustained improvement of calcification propensity score (serum T50) after successful kidney transplantation: Reanalysis of a randomized controlled trial of ibandronate. Clin. Transplant..

[54] Dekker M., Pasch A., Van Der Sande F., Konings C., Bachtler M., Dionisi M., Meier M., Kooman J., Canaud B. (2016). High-Flux Hemodialysis and High-Volume Hemodiafiltration Improve Serum Calcification Propensity. PLoS ONE.

[55] Bristow S., Gamble G.D., Pasch A., O’Neill W.C., Stewart A., Horne A., Reid I.R. (2015). Acute and 3-month effects of calcium carbonate on the calcification propensity of serum and regulators of vascular calcification: Secondary analysis of a randomized controlled trial. Osteoporos. Int..

[56] Dahle D.O., Åsberg A., Hartmann A., Holdaas H., Bachtler M., Jenssen T.G., Dionisi M., Pasch A. (2015). Serum Calcification Propensity Is a Strong and Independent Determinant of Cardiac and All-Cause Mortality in Kidney Transplant Recipients. Arab. Archaeol. Epigr..

[57] Keyzer C.A., De Borst M.H., Berg E.V.D., Jahnen-Dechent W., Arampatzis S., Farese S., Bergmann I.P., Floege J., Navis G., Bakker S.J. (2015). Calcification Propensity and Survival among Renal Transplant Recipients. J. Am. Soc. Nephrol..

[58] Berchtold L., Ponte B., Moll S., Hadaya K., Seyde O., Bachtler M., Vallée J.-P., Martin P.-Y., Pasch A., De Seigneux S. (2016). Phosphocalcic Markers and Calcification Propensity for Assessment of Interstitial Fibrosis and Vascular Lesions in Kidney Allograft Recipients. PLoS ONE.

[59] De Seigneux S., Ponte B., Berchtold L., Hadaya K., Martin P.-Y., Pasch A. (2015). Living kidney donation does not adversely affect serum calcification propensity and markers of vascular stiffness. Transpl. Int..

[60] Smith E.R., Ford M.L., Tomlinson L., Bodenham E., McMahon L.P., Farese S., Rajkumar C., Holt S.G., Pasch A. (2013). Serum Calcification Propensity Predicts All-Cause Mortality in Predialysis CKD. J. Am. Soc. Nephrol..

[61] Zarb Y., Weber-Stadlbauer U., Kirschenbaum D., Kindler D.R., Richetto J., Keller D., Rademakers R., Dickson D.W., Pasch A., Byzova T.V. (2019). Ossified blood vessels in primary familial brain calcification elicit a neurotoxic astrocyte response. Brain.

[62] Kendrick J., Shah P., Andrews E., You Z., Nowak K.L., Pasch A., Chonchol M. (2018). Effect of Treatment of Metabolic Acidosis on Vascular Endothelial Function in Patients with CKD. Clin. J. Am. Soc. Nephrol..

[63] Voelkl J., Tuffaha R., Luong T.T., Zickler D., Masyout J., Feger M., Verheyen N., Blaschke F., Kuro-O M., Tomaschitz A. (2018). Zinc Inhibits Phosphate-Induced Vascular Calcification through TNFAIP3-Mediated Suppression of NF-κB. J. Am. Soc. Nephrol..

[64] Jahnen-Dechent W., Trindl A., Godovac-Zimmermann J., Müller-Esterl W. (1994). Posttranslational Processing of Human alpha2-HS Glycoprotein (Human Fetuin). Evidence for the Production of a Phosphorylated Single-Chain Form by Hepatoma Cells. JBIC J. Boil. Inorg. Chem..

[65] Mathews S.T., Chellam N., Srinivas P.R., Cintron V.J., Leon M., Goustin A.S., Grunberger G. (2000). Alpha2-HSG, a specific inhibitor of insulin receptor autophosphorylation, interacts with the insulin receptor. Mol. Cell. Endocrinol..

[66] Auberger P., Falquerho L., Contreres J.O., Pagès G., Le Cam G., Rossi B., Le Cam A. (1989). Characterization of a natural inhibitor of the insulin receptor tyrosine kinase: cDNA cloning, purification, and anti-mitogenic activity. Cell.

[67] Suzuki M., Shimokawa H., Takagi Y., Sasaki S. (1994). Calcium-binding properties of fetuin in fetal bovine serum. J. Exp. Zoöl..

[68] Herrmann M., Schäfer C., Heiss A., Gräber S., Kinkeldey A., Büscher A., Schmitt M.M., Bornemann J., Nimmerjahn F., Herrmann M. (2012). Clearance of Fetuin-A–Containing Calciprotein Particles Is Mediated by Scavenger Receptor-A. Circ. Res..

[69] Hamano T., Matsui I., Mikami S., Tomida K., Fujii N., Imai E., Rakugi H., Isaka Y. (2010). Fetuin-mineral complex reflects extraosseous calcification stress in CKD. J. Am. Soc. Nephrol..

[70] Smith E.R., Hewitson T., Cai M.M.X., Aghagolzadeh P., Bachtler M., Pasch A., Holt S.G. (2017). A novel fluorescent probe-based flow cytometric assay for mineral-containing nanoparticles in serum. Sci. Rep..

[71] Ismail A.H., Schäfer C., Heiss A., Walter M., Jahnen-Dechent W., Leonhardt S. (2011). An electrochemical impedance spectroscopy (EIS) assay measuring the calcification inhibition capacity in biological fluids. Biosens. Bioelectron..

[72] Ter Braake A.D., Tinnemans P.T., Shanahan C.M., Hoenderop J.G.J., De Baaij J.H. (2018). Magnesium prevents vascular calcification in vitro by inhibition of hydroxyapatite crystal formation. Sci. Rep..

[73] Huang M., Zheng L., Xu H., Tang D., Lin L., Zhang J., Li C., Wang W., Yuan Q., Tao L. (2020). Oxidative stress contributes to vascular calcification in patients with chronic kidney disease. J. Mol. Cell. Cardiol..

[74] Rubin M.R., Silverberg S.J. (2004). Vascular calcification and osteoporosis--the nature of the nexus. J. Clin. Endocrinol. Metab..

[75] Keeley F., Sitarz E. (1985). Identification and quantitation of α2-HS-glycoprotein in the mineralized matrix of calcified plaques of atherosclerotic human aorta. Atherosclerosis.

[76] Binkert C., Demetriou M., Sukhu B., Szweras M., Tenenbaum H.C., Dennis J. (1999). Regulation of osteogenesis by fetuin. J. Boil. Chem..

[77] Ketteler M., Bongartz P., Westenfeld R., Wildberger J.E., Mahnken A.H., Böhm R., Metzger T., Wanner C., Jahnen-Dechent W., Floege J. (2003). Association of low fetuin-A (AHSG) concentrations in serum with cardiovascular mortality in patients on dialysis: A cross-sectional study. Lancet.

[78] Fontaine M.A., Albert A., Dubois B., Saint-Remy A., Rorive G. (2000). Fracture and bone mineral density in hemodialysis patients. Clin. Nephrol..

[79] Kirkpantur A., Altun B., Hazirolan T., Akata D., Arici M., Kirazli S., Turgan C. (2009). Association Among Serum Fetuin-A Level, Coronary Artery Calcification, and Bone Mineral Densitometry in Maintenance Hemodialysis Patients. Artif. Organs.

[80] Pasch A., Jahnen-Dechent W., Smith E.R. (2018). Phosphate, Calcification in Blood, and Mineral Stress: The Physiologic Blood Mineral Buffering System and Its Association with Cardiovascular Risk. Int. J. Nephrol..

